# Supplementary material for: Chromothripsis during telomere crisis is independent of NHEJ, and consistent with a replicative origin
Source: Genome Res. 2019 May;29(5):737–49. doi: 10.1101/gr.240705.118 (PMC6499312; doi:10.1101/gr.240705.118)
Supplement: Supplemental Material [file supp_gr.240705.118_Supplemental_file_1.zip › contigs/annotated_contigs/DB51/contig.2.DB51_length_426_mean_cov_7.98591549296.docx]

**DB51_length_426_mean_cov_7.98591549296**

```
TCTATTAGTTATGGATATTTTACATTTTACATGACTGACTATATTAAGGGATAGGAAAAAAACTTCCTTTGATTAGAGAGAATTCTTTG
 >chr6:63634851-63635059 + E=1e-107 p=3e-02
CATTATGATGACATATTACAGTAAGGGGCTTACTAAAACTCCTCAGCATCTTTGAAAGCCATGCCAGTAGTGGAAAAGGCTAAATTATA

TATGTATCAGGCTCTTTATACTTAGCTTAG|AAAGT|AGTAGAAAGTAGGGGGTGAATATATTGCAGGAAAAGACAATATTATGGTTTA
                                    >chr6:63635796-63636009 + E=1e-116
TTAGTTATCTTTTGCTGAATAAAAAAGTACTACCAACTCAGTGGCTTATAACAACATGCATTAATTATCTCATAACTTCCATGTGTCTG

GGTCTGGGAATGCCTAGCTGAGCCCTGTAGTCAGTGTCTCACAAGCTGTATTCAAGGTGCTGGCTTTGTCTA
```
